# *JACC: Basic to Translational Science* Top Reviewers 2023: With Appreciation and Gratitude

**DOI:** 10.1016/j.jacbts.2023.12.004

**Published:** 2024-01-22

**Authors:** Brian H. Annex, Michael R. Bristow, Nikolaus G. Frangogiannis, Daniel P. Kelly, Maria Kontaridis, Peter Libby, William Robb MacLellan, Coleen A. McNamara, Douglas L. Mann, Geoffrey S. Pitt, Karin R. Sipido

As the new year begins, the editorial team thanks all of our reviewers who have worked tirelessly to maintain the quality and the integrity of the review process for *JACC: Basic to Translational Science*. Given the diverse range of scientific articles that we publish, identifying reviewers with the necessary expertise is a challenging task. In 2023, we were privileged to receive exceptional, timely, and high-quality reviews for the manuscripts submitted for peer evaluation.

The Editors give special recognition to the reviewers who went above and beyond what was asked of them, and to recognize their exemplary efforts here. We recognize the individuals listed below (in alphabetical order) for the timeliness and quality of their reviews. Individuals with an asterisk before their name were also recognized as outstanding reviewers in 2022, and those with a dagger were recognized as outstanding reviewers in 2021 and 2022.

The professional acumen and integrity of the review process contribute enormously to the quality of the scientific content published in our journal. The Editors of *JACC: Basic to Translational Science* recognize that a rigorous and balanced scientific review takes time and effort to perform, which far too often receives insufficient acknowledgement and gratitude. So we here express our deep appreciation and gratitude to all of our reviewers for their abiding efforts to advance the field of cardiovascular translational medicine.

Matthew Alexander

Magnus Bäck

∗Daniel Burkhoff

Abhinav Diwan

Gustavo Egea

Andrew Frelinger

Kiyotake Ishikawa

Brian Jensen

Ha Won Kim

Jonathan Kirk

Attila Kiss

∗Walter Koch

†Issei Komuro

Kory Lavine

†Gary Lopaschuk

Steven Marx

†Lewis Rubin

†Junichi Sadoshima

Katrin Schaefer

Ashish Sharma

Andreas Wagner

Yibin Wang

Kathleen Woulfe

## Footnotes

∗ Top reviewer 2022

† Top reviewer 2021, 2022

